# *Bacillus subtilis* improves antioxidant capacity and optimizes inflammatory state in broilers

**DOI:** 10.5713/ab.23.0320

**Published:** 2024-02-23

**Authors:** Yu Zhang, Junyan Zhou, Linbao Ji, Lian Zhang, Liying Zhao, Yubing Guo, Haitao Wei, Lin Lu

**Affiliations:** 1 Animal Science and Technology College, Beijing University of Agriculture, Beijing, 102206, China; 2 College of Animal Science and Technology, China Agricultural University, Beijing, 100193, China

**Keywords:** Antioxidant, *Bacillus subtilis*, Broiler Chicken, Inflammatory, Meat Quality

## Abstract

**Objective:**

*Bacillus subtilis*, a kind of probiotic with broad-spectrum antibacterial function, was commonly used in livestock and poultry production. Recent research suggested that *Bacillus subtilis* may have antioxidant properties and improve immune response. This study aimed to verify the probiotic function of *Bacillus subtilis* in the production of broiler chickens.

**Methods:**

A total of 324 (1-day-old) Arbor Acres broilers were selected and randomly divided into three groups: basal diet group (Ctr Group), basal diet + antibiotic growth promoter group (Ctr + AGP) and basal diet + 0.5% *Bacillus subtilis* preparation group (Ctr + Bac). The experiment lasted for 42 days. Muscle, serum and liver samples were collected at 42 days for determination.

**Results:**

The results showed that *Bacillus subtilis* could decrease malondialdehyde content in the serum and liver (p<0.05) and increase superoxide dismutase 1 mRNA expression (p<0.01) and total superoxide dismutase (p<0.05) in the liver. In addition, compared with AGP supplementation, *Bacillus subtilis* supplementation increased interleukin-10 (IL-10) and decreased tumor necrosis factor-α and IL-1β level in the serum (p<0.05). At 45 minutes after slaughter Ctr + Bac presented a higher a* value of breast muscle than Ctr Group (p< 0.05), while significant change in leg muscle was not identified. Moreover, there was no difference in weight, shear force, cooking loss and drip loss of breast and leg muscle between treatments.

**Conclusion:**

Our results demonstrate that *Bacillus subtilis* in diet can enhance antioxidant capacity and optimize immune response of broilers.

## INTRODUCTION

Mitochondria convert oxygen and organic molecules into cellular energy during aerobic respiration while generating significant amounts of reactive oxygen species (ROS) [1]. ROS act as signaling molecules and participate in normal physiological processes, including intracellular signaling. For example, ROS induces phosphorylation of AKT serine/threonine kinase 1 at Ser473, leading to the activation of mechanistic target of rapamycin kinase complex 2, which ultimately promotes myofiber cell differentiation [2]; besides, ROS can interact with cysteine residues in protein molecules, leading to conformational changes through sulfhydryl oxidation. However, high concentrations of ROS can have detrimental effects on cells, damaging lipids, proteins, and nucleic acids [3]. Henry et al [4] reported that ROS can directly harm DNA structure and oxidize Rec A recombinase, inhibiting DNA repair and recombination, while Diwanji and Bergmann [5] demonstrated that ROS can induce upregulation and accumulation of matrix metalloproteinase 2 in the basement membrane (BM), causing BM damage.

To prevent excessive ROS accumulation, the body employs various antioxidant mechanisms that can inhibit ROS production or neutralize ROS generated during metabolism [6]. Such as the classical KEAP1-Nrf2-ARE signaling pathway. Disruption of the balance between oxidative response and antioxidant mechanisms can cause oxidative stress, leading to cellular damage [7,8]. Oxidative stress can also result in vascular dysfunction [9], nonalcoholic fatty liver disease [10], obstructive pulmonary disease [11], and other illnesses. Additionally, ROS can induce the release of inflammatory factors by regulating the signaling pathway downstream of pattern recognition receptors, causing chronic sterile inflammation [12].

High-density breeding environments can lead to serious oxidative stress in poultry, which may affect their health. Bacillus exhibits broad-spectrum antibacterial properties [13–15], positioning it as a potential probiotic for animal husbandry applications, and recent studies identified its antioxidant properties, further highlighting its potential for promoting animal health. For instance, Ruan et al [16] used soybean meal as a substrate for *Bacillus subtilis* fermentation, producing proteins and peptides with potent antioxidant activity. Cui et al [17] found that soybean fermentation with Bacillus increased the activity of antioxidant enzymes and reduced malondialdehyde levels in the serum and liver of mice. Furthermore, Rahman et al [18] isolated and purified a polypeptide with a molecular weight of approximately 1.0 kDa from a Bacillus strain that exhibited broad-spectrum antibacterial activity and up-regulated the transcription and translation of NRF-2, thereby enhancing the activity of antioxidant enzymes. It is necessary to investigate the currently unexplored antioxidant function of a screened *Bacillus subtilis* strain with probiotic potential incorporated into the feeding regimen of broilers. Therefore, the current study aims to investigate the effects of feeding a screened *Bacillus subtilis* strain with the probiotic potential to Arbor Acres (AA) broilers on antioxidant function, inflammatory state, and meat quality. The results of the present study will contribute to a better understanding of the potential applications of *Bacillus subtilis* in poultry production.

## MATERIALS AND METHODS

### Animal care

The present experiment was approved by the China Agricultural University Animal Care and Use Committee (Beijing, AW12401202-1-1).

### *Bacillus subtilis* preparation and basal diet

The Ministry of Agriculture Feed Industry Centre screened soil samples from a pig experimental base in Beijing and obtained a probiotic strain through ultraviolet mutagenesis. The strain was identified as *Bacillus subtilis* via 16S RNA sequencing and deposited with the General Microbiology Center (CGMCC) of the Chinese Microbial Species Preservation Administration Committee on July 12, 2021, under the classification name of *Bacillus subtilis* and storage number of CGMCC No. 22855. The laboratory confirmed the strain’s potent antibacterial properties and successfully applied for a patent. The strain was utilized to produce solid fermented feed through probiotic fermentation technology in *Bacillus subtilis* preparations for subsequent experiments. The basic diet used in this study was also provided by the Ministry of Agriculture Feed Industry Centre. The basal diet was a corn-soybean meal type ration, and the composition was as shown in Supplementary Table S1.

### Animals grouping and diets

A total of 324 1-day-old AA broilers with an average body weight of 45±5 g were randomly divided into 3 groups with 18 replicates (cages) per group and 6 AA broilers per replicate according to randomized block design. All replicates were randomly distributed within the experimental area. The blank control group (Ctr Group) was fed with a basal diet, the antibiotic growth promoter (AGP) group (Ctr + AGP) was fed with a basal diet supplemented with chlortetracycline, and the *Bacillus subtilis* preparation group (Ctr + Bac) was fed with a basal diet supplemented with 0.5% *Bacillus subtilis* preparation.

The experiment lasted for 42 days. All broilers were provided with fresh and clean water, as well as the freedom to access food. From days 1 to 7, the temperature was maintained at 32°C to 35°C. From days 8 to 14, the temperature was kept between 29°C to 32°C. Between days 15 to 42, the temperature was maintained at 25°C to 29°C. All broilers were vaccinated with the Newcastle disease (ND) and infectious bronchitis (IB) vaccine and infectious bursal disease (IBD) vaccine at 7 and 14 days of age, respectively. Throughout the experiment, supplemental light was provided for 24 hours in the experimental area. On day 42, one broiler was selected from each replicate to collect experimental samples.

### Sample collection

On day 41, all feed was emptied at 8:00 PM, and blood samples were collected from the sub-wing vein at 8:00 AM on day 42. The blood samples were allowed to stand at 4°C for 2 hours before being stratified. The samples were then placed in a low-temperature, high-speed centrifuge and centrifuged at 4°C and 3,500 r/min for 10 minutes. The upper layer of serum was absorbed using a pipetting gun, transferred to a blank centrifuge tube, and stored at −80°C for future use.

After blood collection via cardiac puncture, the chickens were sacrificed. Liver tissues, approximately 2 cm in length, 0.8 cm in width, and 1 cm in height, were collected from the tip of the liver lobule for histological observation. The liver tissues were fixed with a 4% paraformaldehyde solution. Additionally, the remaining liver tissue was collected, and excess blood on the surface was cleaned with sterile normal saline. The liver tissue was then transferred to sterile frozen tubes and stored in an ultra-low refrigerator until ready for use. Throughout the sampling process, efforts were made to ensure that liver samples were collected from the same location for each broiler.

### Meat quality

Meat quality indicators for the breast and leg muscles, including meat color, pH value, cooking loss, and drip loss, were assessed. Following the slaughter of the broilers, the left breast and leg muscles were extracted and placed on an enamel plate for 45 minutes. Three randomly selected spots on the muscle were then used to determine the pH value and meat color (lightness [L*], redness [a*], and yellowness [b*]) using a pH meter and digital color reader. Subsequently, the samples were stored at 4°C for 24 hours before being retested.

The right breast and leg muscles of the broilers were collected, and the meat sample was cut into strips measuring 2 cm×5 cm×3 cm. The initial weight of the sample (W1) was recorded after weighing. The muscle fiber was then hooked with an iron wire, allowing it to hang vertically down in an aerated polyethylene film bag. The bag was tightly tied at the mouth to prevent contact between the muscle sample and the bag wall. The sample was hung in a refrigerator at 4°C for 24 hours. After 24 hours, the muscle samples were weighed again and the final weight (W2) was recorded. Subsequently, the drip loss of muscle samples was calculated using Equation 1.


Equation 1 $$Drip\;loss=\frac{W1-W2}{W1}\times 100\%$$

Samples weighing approximately 100 g were extracted from the remaining right pectoral and leg muscles and weighed, with the initial body weight recorded as W3. The muscle samples were then placed in a thermostatic water bath and processed at 90°C for 45 minutes. Once processed, the muscle sample was removed from the water bath and allowed to cool to room temperature. Excess water on the surface of the sample was wiped off with absorbent paper and left to dry. The muscle was then weighed again and the final weight was recorded as W4. Subsequently, the cooking loss of muscle samples was calculated using Equation 2.


Equation 2 $$Cooking\;loss=\frac{W3-W4}{W3}\times 100\%$$

### Determination of antioxidant index

The serum samples and liver samples stored at −80°C were removed and thawed on ice. Normal saline was added to the thawed liver tissue at a weight ratio of 1:9 at 4°C. The resulting mixture was then ground to uniformity at 4°C and 60.00 HZ using an automatic sample freezing grinder. The ground samples were subsequently centrifuged at 3,000 R/min at 4°C for 15 min in a cryogenic high-speed centrifuge. The resulting supernatant was 10% tissue homogenate, which was transferred to a blank centrifuge tube using a pipette gun for subsequent tests. Enzyme activity of glutathione peroxidase (GSH-Px) and total superoxide dismutase (T-SOD), as well as the content of malondialdehyde (MDA) in serum and 10% liver tissue homogenate, were detected using the GSH-Px assay kit (colorimetric method), total superoxide dismutase assay kit (hydroxylamine method), and MDA assay kit, respectively.

### Real-time fluorescence quantitative reverse transcription polymerase chain reaction

Total RNA was extracted from liver tissues using the M5 HiPer RNApure Universal Animal ultra-pure total RNA Rapid Extraction Kit (Mei5 Biotechnology Co., Ltd., Beijing, China). The liver tissue samples were then reverse-transcribed into cDNA using the M5 Super qPCR RT kit with gDNA remover (Mei5 Biotechnology Co., Ltd., China). The resulting cDNA was used as the template for real-time fluorescence quantitative reverse transcription polymerase chain reaction (qRT-PCR). SOD1 and SOD3 were used as the target gene and glyceraldehyde-3-phosphate dehydrogenase (GAPDH) was used as the housekeeping gene. The primers used in the qRT-PCR were provided by Tsingke Biotechnology Co., Ltd, Beijing, China, and the TSINGKE TSE202 2×T5 Fast qPCR Mix (SYBR Green I) was used for the qRT-PCR. Table 1 shows detailed information on the primers used in the experiment. The test results were analyzed using the 2^−ΔΔCt^ method and each experiment was repeated three times.

### Determination of inflammatory cytokines

The contents of tumor necrosis factor alpha (TNF-α), interleukin-1β (IL-1β), and IL-10 in serum and 10% liver tissue homogenate were detected using the TNF-α assay kit (enzyme immunoassay), IL-1β assay kit (enzyme immunoassay), and IL-10 assay kit (enzyme immunoassay), respectively.

### Histological observation of liver tissue

The liver samples were retrieved from a 4% paraformaldehyde solution, embedded in paraffin, and sectioned into tissue sections of 3 μm thickness using a rotary microtome. Hematoxylin and eosin dyes (H&E) were applied to the tissue sections, which were then observed under a high-magnification microscope to assess the morphological changes in the liver tissue.

### Statistical analysis

One-way analysis of variance was used to analyze the differences between groups using IBM SPSS Statistics version 23. Statistical significance is considered to be extremely significant if p<0.01, significant if p<0.05, and to have a trend towards statistical significance if 0.05<p<0.1. The statistics and other plots were completed with GraphPad Prism 9 software (Version 9.3.1, USA).

## RESULTS

### Effects of *Bacillus subtilis* preparation on antioxidant capacity of broilers

**Figure 1:**
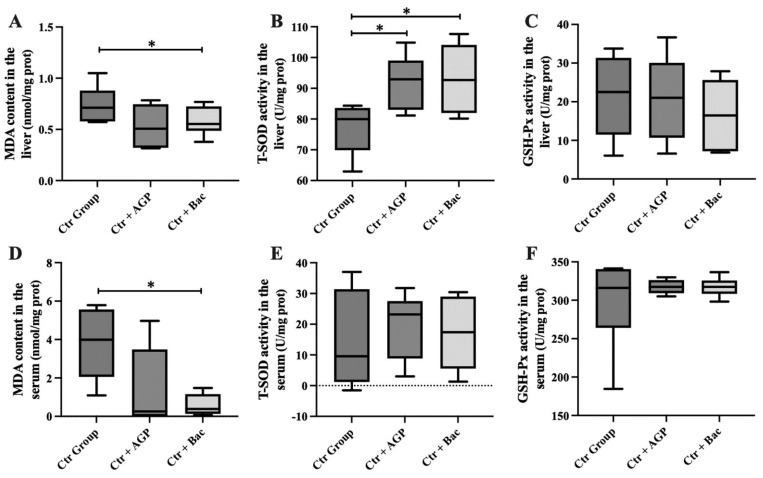
*Bacillus subtilis* preparation can reduce oxidative stress injury and improve the activity of antioxidant enzymes of broiler. (A) The MDA content, (B) the activity of T-SOD, (C) and the activity of GSH-Px in the liver; (D) the MDA content, (E) the activity of T-SOD, (F) and the activity of GSH-Px in the serum are presented. MDA, malondialdehyde; T-SOD, total superoxide dismutase; GSH-Px, glutathione peroxidase. *Significant differences between the group means p<0.05.

The results indicate that the MDA content in the liver and serum of the Ctr + Bac group was significantly lower than those of the Ctr group (p<0.05; Figure 1). Compared with the Ctr group, the T-SOD activity in the liver of Ctr + AGP and Ctr + Bac groups was dramatically increased (p<0.05). Additionally, there was no significant difference in GSH-Px activity in the liver and serum and T-SOD in the serum between treatments.

### Effect of *Bacillus subtilis* preparation on mRNA expression of SOD1 and SOD3 in liver

**Figure 2:**
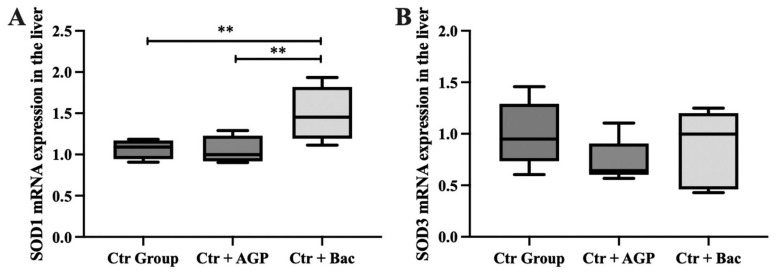
*Bacillus subtilis* preparation can improve the expression of antioxidant enzymes in the liver of broilers. The mRNA expression of SOD1 (A) and SOD3 (B) are presented. SOD1, superoxide dismutase 1; SOD3, superoxide dismutase 3. *, ** Significant differences between the groups at p<0.05 and p<0.01, respectively.

The GAPDH transcription level in the liver was used as a reference to analyze changes in the transcription level of the *SOD1* and *SOD3* genes in the liver of the three experimental groups. The effects of *Bacillus subtilis* preparation and AGP on SOD1 and SOD3 transcription levels in the liver tissue of broilers are shown in Figure 2. The transcription level of SOD1 in the liver tissue of the Ctr + Bac group was significantly higher than that of both the Ctr Group and the Ctr + AGP group (p<0.01). Furthermore, there were no significant differences observed in the liver SOD3 expression among the treatment groups.

### Effect of *Bacillus subtilis* preparation on liver tissue morphology

**Figure 3:**
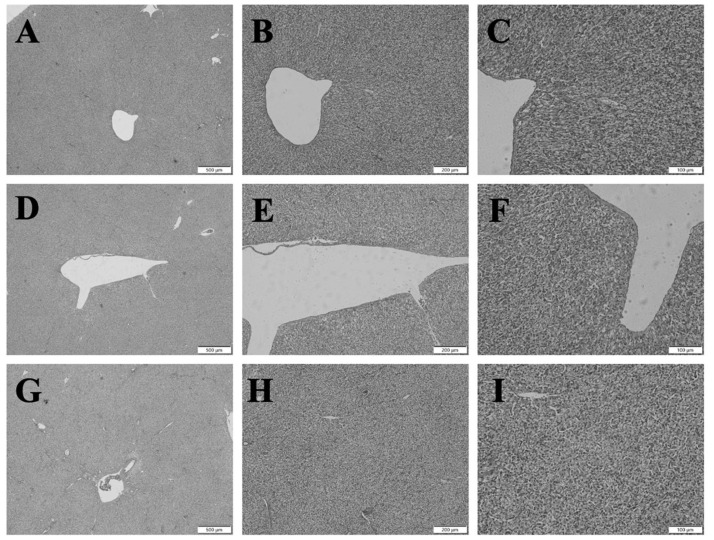
Effect of *Bacillus subtilis* preparation on the liver morphology of broilers, the liver sections were stained with hematoxylin and eosin. (A) Liver tissue sections of Ctr Group at 40× magnification. (B) Liver tissue sections of Ctr Group at 100× magnification. (C) Liver tissue sections of Ctr Group at 200× magnification. (D) Liver tissue sections of Ctr + AGP group at 40× magnification. (E) Liver tissue sections of Ctr + AGP group at 100× magnification. (F) Liver tissue sections of Ctr + AGP group at 200× magnification. (G) Liver tissue sections of Ctr + Bac group at 40× magnification. (H) Liver tissue sections of Ctr + Bac group at 100× magnification. (I) Liver tissue sections of Ctr + Bac group at 200× magnification. Ctr, control; AGP, antibiotic growth promoter.

To investigate whether *Bacillus subtilis* preparation could induce liver damage in broilers, histopathological observations were performed. The results are shown in Figure 3, which indicate that the liver tissue structure of the Ctr group, Ctr + Bac, and Ctr + AGP groups was intact, with clear hepatic lobules, closely arranged hepatocytes, and an obvious hepatocyte cable structure. No obvious hepatic lesions or injuries were observed in the visual field.

### Effect of *Bacillus subtilis* preparation on inflammatory factors

**Figure 4:**
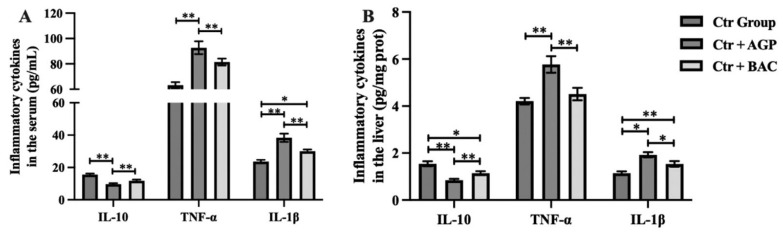
*Bacillus subtilis* preparation can modulate the inflammatory state of the body. (A) Effect of *Bacillus subtilis* preparation on the level of medium inflammatory factors (IL-10, TNF-α, and IL-1β) in the liver and (B) serum. IL-10, interleukin-10; TNF-α, tumor necrosis factor-α. *, ** Significant differences between the groups at p<0.05 and p<0.01, respectively.

Changes in antioxidant function are often accompanied by changes in the inflammatory state. To investigate the effect of *Bacillus subtilis* preparation on the upregulation of antioxidant capacity and its influence on the inflammatory state, the levels of IL-1β, IL-10, and TNF-α in the liver and serum of broilers in the three groups were detected, as shown in Figure 4. Compared with the Ctr group, Ctr + Bac group significantly increased IL-1β (p<0.05) levels in the serum and IL-10 (p<0.05) and IL-1β (p<0.01) level in the liver. Moreover, Ctr + Bac group and Ctr group showed higher IL-10 levels and lower TNF-α and IL-1β in the liver and serum than the Ctr + AGP group (p<0.05).

### Effects of *Bacillus subtilis* preparation on meat quality of broilers

**Figure 5:**
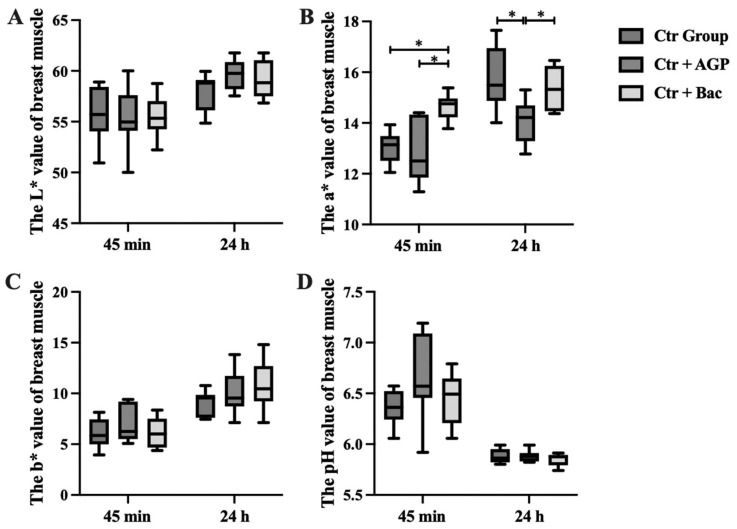
Effect of *Bacillus subtilis* preparation on meat color and pH of breast muscle. The (A) L* value, (B) a* value, (C) b* value and (D) pH value of breast muscle are presented. * Significant differences between the group means p<0.05.

**Figure 6:**
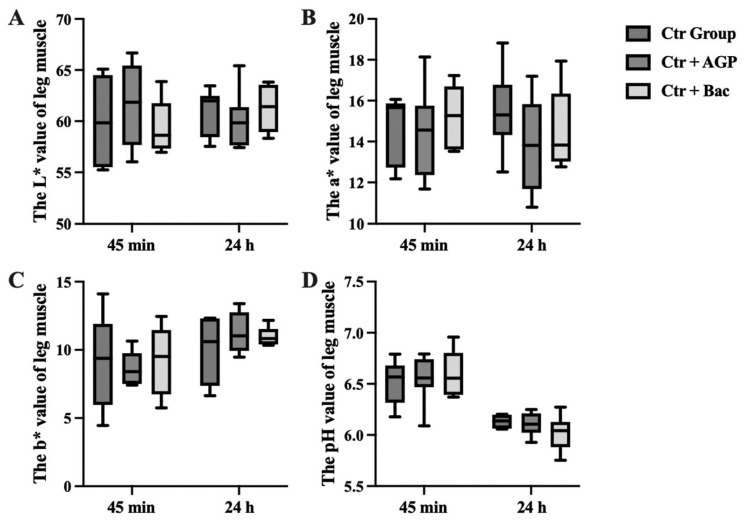
Effect of *Bacillus subtilis* preparation on meat color and pH of leg muscle. The (A) L* value, (B) a* value, (C) b* value and (D) pH value of leg muscle are presented.

**Figure 7:**
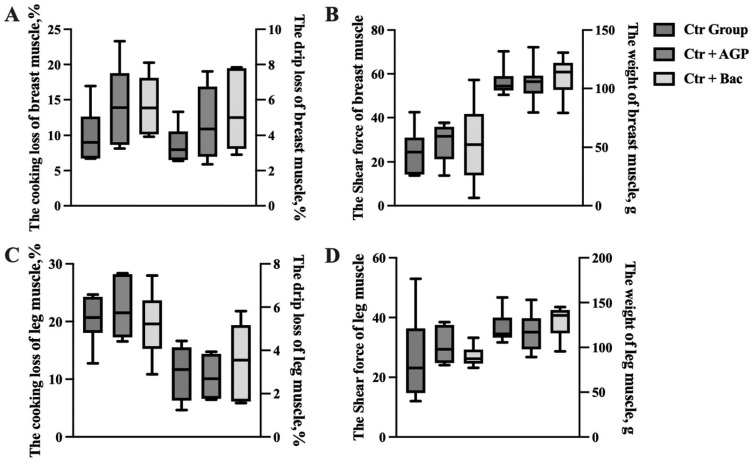
Effect of *Bacillus subtilis* preparation on meat quality of breast and leg muscle. The (A) cooking loss and drip loss of breast muscle, (B) the shear force and weight of breast muscle, (C) the cooking loss and drip loss of leg muscle and (D) the shear force and weight of leg muscle are presented.

At 45 minutes after slaughter, the a* value of breast muscle in the Ctr + Bac group was significantly higher than that in both the Ctr group and the Ctr + AGP group (p<0.05). In addition, at 24 hours after slaughter, the Ctr + AGP group showed significantly lower a* values than the other two groups (p<0.05). Moreover, the b* value of breast muscle in the Ctr + Bac group was markedly increased compared with the Ctr group (p<0.05). The results are shown in Figure 5 and Figure 6. There was no observable difference in meat color and pH of leg muscle and weight, shear force, cooking loss, and drip loss of breast and leg muscle between treatments (Figure 7).

## DISCUSSION

### Effect of *Bacillus subtilis* on the antioxidant function of broilers

The presence of stress in commercial broiler production can have a negative impact on their productivity and reproductive performance. Scientific research has established that in poultry production, stressors from different sources (such as technology, environment, and nutrition) can trigger oxidative stress at the cellular level [19]. This phenomenon arises due to the surplus generation of free radicals or deficient antioxidant defense mechanisms [20]. Therefore, the antioxidant defense mechanisms assume a crucial function in ameliorating the detrimental consequences of free radicals on critical biological macromolecules, including proteins, lipids, and DNA, which could profoundly impact the performance of poultry production. Recent studies have found that bacillus supplements can not only regulate intestinal flora homeostasis in animals but also enhance antioxidant capacity, which may be a potential antioxidant additive for poultry production [21,22]. The present results suggested that *Bacillus subtilis* can enhance antioxidant performance by increasing T-SOD levels and optimize the immune response of AA broilers.

MDA is a highly reactive, mutagenic, and tumorigenic three-carbon dialdehyde that is produced during the peroxidation of polyunsaturated fatty acids and the metabolism of arachidonic acid [23]. MDA can react with DNA to form MDA-DNA adducts, making it a valuable biomarker of endogenous DNA damage. The monitoring of MDA levels in various biological systems can serve as a critical indicator of lipid peroxidation in both *in vitro* and *in vivo* settings and has been linked to various health disorders [24]. SOD is a metal-containing antioxidant enzyme present *in vivo*. It catalyzes the dismutation of superoxide anion radicals into oxygen and hydrogen peroxide, thereby playing a vital role in maintaining the balance between oxidative stress and antioxidant defense [25]. SOD1 is ubiquitously distributed in the nucleus, cytoplasm, and extracellular space, and is widely regarded as the principal and preeminent SOD, operating cooperatively with the antioxidant enzyme glutathione peroxidase to eliminate detrimental ROS within the cell [26]. In this study, MDA content in liver and serum was significantly lower in the Ctr + Bac group than in the Ctr group, suggesting that *Bacillus subtilis* was able to reduce oxidative stress injury. We also assayed GSH-Px and SOD enzyme activities in liver and serum, and the results suggested to us that *Bacillus subtilis* exerts its antioxidant function mainly by up-regulating SOD enzyme activities. Based on this clue, we examined the transcript levels of the two major SOD enzymes (SOD1 and SOD2) in the liver. The qRT-PCR results showed that the transcript level of SOD1 in the liver of the Ctr + Bac group was higher than that of the Ctr group and the Ctr + AGP group, which indicated that *Bacillus subtilis* was able to up-regulate hepatic SOD1 mRNA expression as a way to enhance the antioxidant function, and the effect was better than that of AGP.

### Effect of *Bacillus subtilis* on the inflammatory response of broiler chickens

Changes in the redox state often accompany changes in the inflammatory state [27]. To investigate the effect of *Bacillus subtilis* preparations on improving antioxidant function and its impact on the inflammatory state, we measured the levels of three inflammatory factors, IL-1β, IL-10, and TNF-α in the liver and serum. IL-1β [28] and TNF-α [29] are known to promote the inflammatory response, while IL-10 [30] has been thought to inhibit it. However, recent research challenges this simplistic view. For example, IL-10 can promote the growth and differentiation of B cells, leading to autoimmune system diseases [31]. Structurally, Saxton et al [32] have also shown that IL-10 promotes inflammatory responses. The research by Coleman Jr et al [33] suggests that IL-1β can enhance the immune function of the body by forming a heterogeneous complex with high mobility group box 1. Similarly, TNF-α has immunosuppressive properties [34], binding to TNF receptor type 2 to activate regulatory T cells, thereby regulating the body’s immune function [35]. These findings indicate that we cannot simply categorize inflammatory factors as pro-inflammatory or anti-inflammatory, nor can we evaluate the body’s health status solely based on changes in the content of inflammatory factors. To investigate this further, we performed histological sectioning and HE staining on broiler liver tissue and observed tissue morphology under high and low magnification. No significant differences or lesions were found in the liver tissue of the three experimental groups of broilers, indicating that the *Bacillus subtilis* supplementation and the resulting changes in antioxidant function and immune response did not have any negative impact on the health of the organism. The change in inflammatory state is likely to be a positive immune response of the body. From the mechanism of action analysis, Bacillus are capable of secreting a variety of lipopeptide secondary metabolites, such as surfactins, iturins, and fengycins. It has been reported that these secondary metabolites have a variety of biological activities, such as modulation of intestinal flora and host inflammatory response. Surfactin reduces the abundance of colitis-associated flora [36] and inhibits the inflammatory effects triggered by lipopolysaccharide macrophage interactions [37].

### Effect of *Bacillus subtilis* on meat quality of broilers

The visual appearance is a crucial quality attribute of poultry meat, as consumers tend to associate it with the product’s freshness, and their purchase decision is often based on their perception of its appeal [38]. Previous research has shown that the concentration of different redox states of myoglobin in muscle is a crucial factor affecting the color of meat [39]. Myoglobin, a binding protein composed of a peptide chain and a heme prosthetic group, plays a role in storing and transporting oxygen in muscle tissue [40]. In the absence of oxygen, myoglobin is mainly present in the deoxygenated state, known as deoxymyoglobin (DeoMb) [41]. If muscle tissue contains high levels of DeoMb, it appears purplish-red in color. When DeoMb comes into contact with oxygen, the heme prosthetic group can bind to oxygen in an oxygenated form instead of being oxidized [42], resulting in the conversion of DeoMb to oxymyoglobin (OxyMb) and giving the meat a bright red color [43]. Additionally, Fe2+ in the heme prosthetic group can be oxidized by oxygen to Fe3+, converting OxyMb to methemoglobin (MetMb) and causing the meat to turn brown [44]. As meat ages, the myoglobin in it is converted to its oxidized form, MetMb, which causes the meat to turn brown. This is a primary reason why consumers reject meat and meat products. The rate of Met Mb formation increases with lipid oxidation, which in turn acts as a catalyst for further lipid oxidation, leading to color and flavor deterioration of the product [45]. In the current study, it was observed that the use of *Bacillus subtilis* resulted in an improvement of broiler meat color as indicated by an increase in the a* value of breast muscle post-slaughter, which could be partly attributed to its antioxidant activity [46]. Additionally, the present study also identified another drawback of AGP, specifically the decrease in breast muscle a* value of broiler.

### Prospects for the application of *Bacillus subtilis*

Currently, there is an urgent need for the breeding industry to find suitable alternatives to antibiotics due to a series of serious consequences of antibiotic misuse. Among the many directions, probiotics are a popular area of research. Probiotics can be developed into probiotic preparations, feed additives and even fermented feeds, which not only maintain the health of livestock and poultry in many ways and improve production and reproduction performance, but also do not bring negative impacts like antibiotics. This study indicates that as research continues, the potential benefits of probiotics will continue to be explored for future applications in the breeding industry.

## CONCLUSION

*Bacillus subtilis* supplementation used in the current experiment can enhance antioxidant function, modulate inflammatory state and improve the meat color of broiler. This study has important implications, as probiotics, particularly those with antioxidative properties, can have a significant impact on enhancing the health and meat quality of poultry in high-density breeding environments.

## Figures and Tables

**Table 1:** Sequences of the primers used for real-time fluorescence quantitative reverse transcription polymerase chain reaction

Genes	Forward primer (5–3′)	Reverse primer (5–3′)
*SOD1*	GGTGCTCACTTCAATCCTGA	TACTTCTGCCACTCCTCCCT
*SOD3*	TCTTGGTTGCCTCCGTCCCT	AGCACTTCGTCTCCACTCCC
*GAPDH*	TCAAATGGGCAGATGCAGGT	GATGGCATGGACAGTGGTCA

*SOD1*, superoxide dismutase 1; *SOD3*, superoxide dismutase 3; *GAPDH*, glyceraldehyde-3-phosphate dehydrogenase.

## SUPPLEMENTARY MATERIAL

**Supplementary Table S1.** A standard corn-soybean meal based diet formula of broiler chickens
