# Supplementary material for: Bacillus subtilis improves antioxidant capacity and optimizes inflammatory state in broilers
Source: Anim Biosci. 2024 Feb 23;37(6):1041–52. doi: 10.5713/ab.23.0320 (PMC11065946; doi:10.5713/ab.23.0320)
Supplement: Supplementary file 1 [file ab-23-0320-Supplementary-Table-1.pdf]

Bacillus Subtilis Improves Antioxidant Capacity and Optimizes Inflammatory State in Broilers

Yu Zhang 1,2,a, Junyan Zhou 1,a, Linbao Ji2, Lian Zhang2, Liying Zhao1, Yubing Guo1, Haitao Wei1, and Lin Lu1,*

Animal Bioscience

Supplementary Table S1
A standard corn-soybean meal based diet formula of broiler chickens.

| Composition | Diet from day 1 to 42 |
|---|---|
| Corn(%) | 59.90 |
| Soybean meal (%) | 22.57 |
| Rape seed cake (%) | 2.00 |
| Distillers Dried Grains with Soluble(%) | 3.50 |
| Corn germ meal(%) | 7.50 |
| Soybean oil (%) | 0.80 |
| Limestone(%) | 1.80 |
| CaHPO4 (%) | 1.00 |
| NaCl (%) | 0.30 |
| L-Lys (%) | 0.16 |
| DL-Met (%) | 0.12 |
| Choline chloride (60%) | 0.10 |
| Multidimensional (%) | 0.04 |
| Mineral addition (%) | 0.15 |
| Phytase (%) | 0.02 |
| Compound enzymes (%) | 0.02 |
| Zinc bacitracin (%) | 0.02 |
| ME (MJ/kg) | 11.75 |
| Crude protein (%) | 18.30 |
| Calcium (%) | 1.00 |
| Total phosphorus (%) | 0.60 |
| Non-phytic acid hosphor | 0.30 |
| Crude fiber (%) | 2.93 |
| Lysine (%) | 0.96 |
| Methionine (%) | 0.41 |
